# Diagnostic Accuracy of Multidetector CT in Detection of Early Interstitial Lung Disease With Its Role in Characterization

**DOI:** 10.7759/cureus.8253

**Published:** 2020-05-23

**Authors:** Shadab Ahmed, Sachin Khanduri, Mushahid Husain, Ahmad Umar Khan, Anchal Singh, Mridul Rajurkar, Syed Zain Abbas, Nazia Khan

**Affiliations:** 1 Radiodiagnosis, Era’s Lucknow Medical College and Hospital, Lucknow, IND; 2 Radiology, Era's Lucknow Medical College and Hospital, Lucknow, IND; 3 Radiodiagnosis, Era's Lucknow Medical College and Hospital, Lucknow, IND

**Keywords:** interstitial lung disease, mdct, emphysema, tuberculosis

## Abstract

Background

Multidetector CT (MDCT) has emerged as a useful option for early diagnosis of interstitial lung disease (ILD) with adequate accuracy.

Methods

A total of 80 patients with restricted pulmonary functions and clinical suspicion of ILD were enrolled in the study. MDCT evaluation was done using Siemens Somatom Force 384 slice multidetector computer tomography machine. Pattern analysis for reticular opacities, nodules and lung opacities was done to reach at a diagnosis. Final diagnosis was based on correlation of radiological and clinicopathological findings. Diagnostic efficacy of MDCT was evaluated in terms of sensitivity, specificity, positive predictive value (PPV), negative predictive value (NPV) and accuracy for detection of ILD.

Results

Mean age of patients was 58 ± 8.75 years. Majority were females (51.3%). History of chronic obstructive pulmonary disease (COPD), tuberculosis and bronchial asthma was revealed in 31 (38.8%), 26 (32.5%) and 16 (20%) patients, respectively. There were 30 (37.5%) patients having no history of respiratory diseases. MDCT diagnosed ILD in 45 (56.3%) cases. On final diagnosis, ILD was diagnosed in 35 (43.8%) cases (15 usual interstitial pneumonia [UIP], 9 cryptogenic organizing pneumonia [COP], 8 nonspecific interstitial pneumonia [NSIP] and 3 respiratory bronchiolitis associated interstitial lung disease [RBILD]). The sensitivity, specificity, positive predictive value, negative predictive value and accuracy of MDCT in detection of ILD was 91.4%, 71.1%, 71.1%, 91.4% and 80%, respectively.

Conclusion

MDCT as a single modality had a high sensitivity for detection of ILD and could be recommended as first line diagnostic imaging technique.

## Introduction

Interstitial lung diseases, also known as diffuse parenchymal lung diseases (DPLD), are a group of disorders involving the distal lung parenchyma [1,2]. Well over one hundred different forms of interstitial lung disease (ILD) have been described [3].

As there are numerous forms of ILD such as idiopathic pulmonary fibrosis (IPF), non-IPF forms of idiopathic interstitial pneumonia (IIP), connective tissue disease associated ILD (CTD-ILD) and hypersensitivity pneumonitis (HP) which can have similar clinical presentations, patients with suspected ILD must undergo an evaluation that adequately establishes a confident diagnosis of a specific ILD as treatment and various management decisions are diagnosis-specific and may vary considerably according to the specific form of ILD that is diagnosed.

Although the combination of history, physical examination, chest X-ray, and other appropriate laboratory testing (peripheral blood tests and lung physiologic testing) may provide a likely diagnosis, additional testing is usually needed to reach a confident diagnosis of a specific ILD. High-resolution CT (HRCT) of the thorax can provide invaluable information that strongly supports a specific diagnosis and may be diagnostic (e.g., typical changes of Usual Interstitial Pneumonia) such that further testing with bronchoscopy or surgical lung biopsy is not required. Indeed, the HRCT has become a standard test for the evaluation of patients with possible ILD [4,5]. Recent studies have shown that multidetector CT (MDCT) thorax helps in better detection, assessment of distribution, evaluation of extent, and characterization of different findings in ILDs, hence increasing the confidence in the diagnosis [6,7]. Hence, the present study was planned to evaluate the role of MDCT in interstitial lung disease at a tertiary care centre in Lucknow.

## Materials and methods

The study was carried out at the Department of Radiodiagnosis in collaboration with the Department of Pulmonary Medicine, Era's Lucknow Medical College, Lucknow over a period starting from January 2016 to June 2017 after obtaining clearance from Institutional Ethics Committee and obtaining informed consent from the patients. A total of 80 patients age >30 years and both the genders who were clinically diagnosed/suspicious of interstitial lung disease having pulmonary function tests indicative of restrictive/obstructive pattern were enrolled in the study. The study was planned as a descriptive study and sample size was calculated as 74 at 90% confidence and 10% error allowance. However, after adding for contingency the sample size was projected at 80.

All the patients were then subjected to radiological evaluation. MDCT evaluation was done using Siemens Somatom Force 384 slice multidetector computer tomography machine.

A pattern-based approach was followed for the purpose of diagnosis. Four patterns were investigated during MDCT evaluation: 1) Reticular Opacities which included interlobular septal thickening (smooth, nodular), irregular, predominant reticular opacities (for pulmonary edema, lymphatic spread of tumor, sarcoidosis, Erdheim-Chester disease or lymphoid pulmonary lesions), traction bronchiectasis and honeycombing; 2) Nodules which included perilymphatic, random and centrilobular; 3) Increased lung opacities which included acute and chronic consolidation/ground glass opacities/ mosaic attenuation; 4) Decreased lung opacities which included cystic disease, cystic bronchiectasis, emphysema and respiratory bronchiolitis. On the basis of presence of these features, a provisional diagnosis was prepared as described by Elicker et al. [8].

Correlation with clinicopathological diagnosis was done in order to achieve the final diagnosis.

Data so collected was analyzed using Statistical Package for Social Sciences (SPSS) version 21.0 (IBM Corp., Armonk, NY). Diagnostic efficacy of MDCT was evaluated in terms of sensitivity, specificity, positive predictive value, negative predictive value and accuracy. The level of agreement of MDCT with final diagnosis was evaluated using Kappa statistic.

## Results

Age of patients ranged from 38 to 75 years. Mean age of patients was 58 ± 8.75 years. Majority of patients were females (51.3%). Smoking history was revealed by 34 (42.5%) patients. Breathlessness (n = 30; 37.5%) was the most common chief complaint followed by dyspnea (n = 22; 27.5%), shortness of breath (25%) and progressive dyspnea (n = 8; 10%), respectively. On enquiry, history of chronic obstructive pulmonary disease (COPD), tuberculosis and bronchial asthma was revealed in 31 (38.8%), 26 (32.5%) and 16 (20%) patients, respectively. There were 30 (37.5%) patients having no history of respiratory diseases (Table 1).

**Table 1:** Demographic profile and clinical characteristics of patients SN: Serial number; SD: Standard deviation; COPD: Chronic obstructive pulmonary disease.

SN	Finding	Statistic
1.	Mean age ± SD (Range) in years	58.00 + 8.75 (38-75)
2.	Male:Female	39 (48.8%): 41 (51.3%)
3.	Smoking history	34 (42.5%)
4.	Chief complaint	
	Breathlessness	30 (37.5%)
	Dyspnea	22 (27.5%)
	Progressive dyspnea	8 (10.0%)
	Shortness of breath	20 (25.0%)
5.	History of respiratory illnesses	
	COPD	31 (38.8%)
	Tuberculosis	26 (32.5%)
	Bronchial asthma	16 (20.0%)
	None	30 (37.5%)

On chest X-ray, majority of patients had honeycombing pattern at lower zone (n = 48; 60%) followed by those showing patchy air space consolidation (25%), reticular opacity at lower zone (n = 8; 10%) and diffuse ground glass opacities (n = 4; 5%). MDCT revealed ground glass opacity in all the cases. Apart from which traction bronchiectasis was the most common finding (n = 73; 91.3%) followed by consolidation (n = 49; 61.3%), interlobular septal thickening (n = 27; 33.8%), reticular opacity (n = 18; 22.5%) and honey combing and apico-basal gradient (n = 8; 10% each). On the basis of MDCT, diagnosis of ILD was made in 45 (56.3%) cases. Among cases with ILD, the most common diagnosis was usual interstitial pneumonia (UIP; n = 17; 21.3%) followed by cryptogenic organizing pneumonia (COP; n = 13; 16.3%), nonspecific interstitial pneumonia (NSIP; n = 8; 10%), acute interstitial pneumonia (AIP; n = 4; 5%) and respiratory bronchiolitis associated interstitial lung disease (RBILD; n = 3; 3.8%), respectively. Among non-ILD cases, 32 (40%) were diagnosed as emphysema and three (3.8%) as tuberculosis (Table 2).

**Table 2:** Imaging findings and MDCT diagnosis MDCT: Multidetector CT; AIP: Acute interstitial pneumonia; COP: Cryptogenic organizing pneumonia; UIP: Usual interstitial pneumonia; RBILD: Respiratory bronchiolitis interstitial lung disease; NSIP: Nonspecific interstitial pneumonia.

SN	Finding	Statistic
1.	Chest X-ray findings	
	Diffuse ground glass opacities	4 (5.0%)
	Honeycombing pattern at lower zone	48 (60.0%)
	Patchy air space consolidation	20 (25.0%)
	Reticular opacity at lower zone	8 (10.0%)
2.	MDCT Findings	
	Ground glass opacity	80 (100%)
	Reticular opacity	18 (22.5%)
	Interlobular septal thickening	27 (33.8%)
	Traction bronchiectasis	73 (91.3%)
	Honey combing	8 (10.0%)
	Apico-basal gradient	8 (10.0%)
	Consolidation	49 (61.3%)
3.	Nodular characteristics on MDCT	
	Perilymphatic	4 (5.0%)
	Random	25 (31.3%)
	Centrilobular	51 (63.8%)
4.	MDCT Diagnosis	
	(a) Interstitial Lung Disease (ILD)	45 (56.3%)
	AIP	4 (5.0%)
	COP	13 (16.3%)
	UIP	17 (21.3%)
	RBILD	3 (3.8%)
	NSIP	8 (10.0%)
	(b) Non-interstitial Lung Disease (Non-ILD)	35 (43.7%)
	Emphysema	32 (40.0%)
	Tuberculosis	3 (3.8%)

Final diagnosis based on clinicopathological correlation revealed ILD in 35 (43.8%) cases only. On final diagnosis, among ILD cases, UIP (n = 15; 18.8%) was the most common diagnosis followed by COP (n = 9; 11.3%), NSIP (n = 8; 10%) and RBILD (n = 3; 3.8%), respectively. Among non-ILD cases (n = 45), emphysema was diagnosed in 34 (42.5%) cases followed by tuberculosis (n = 7; 8.8%) and acute respiratory distress syndrome (ARDS) (n = 4; 5%), respectively (Table 3).

**Table 3:** Final diagnosis based on clinicopathological correlation COP: Cryptogenic organizing pneumonia; UIP: Usual interstitial pneumonia; RBILD: Respiratory bronchiolitis interstitial lung disease; NSIP: Nonspecific interstitial disease; ARDS: Acute respiratory distress syndrome.

SN	Diagnosis	Statistic
1.	Interstitial Lung Disease (ILD)	35 (43.8%)
	COP	9 (11.3%)
	UIP	15 (18.8%)
	RBILD	3 (3.8%)
	NSIP	8 (10.0%)
2.	Non-interstitial Lung Disease (Non-ILD)	45 (56.3%)
	Emphysema	34 (42.5%)
	Tuberculosis	7 (8.8%)
	ARDS	4 (5.0%)

On evaluating the performance of MDCT against final diagnosis for detection of ILD, 32 cases were true positive, 13 were false positive, three were false negative and 32 were true negative. Correspondingly, the sensitivity, specificity, positive predictive value, negative predictive value and accuracy of MDCT in detection of ILD was 91.4%, 71.1%, 71.1%, 91.4% and 80%, respectively. The level of agreement between MDCT and final diagnosis was substantial (k = 0.61; p < 0.001) (Table 4).

**Table 4:** Diagnostic efficacy of MDCT for diagnosis of ILD MDCT: Multidetector computed tomography; ILD: Interstitial lung disease.

MDCT Diagnosis	Final Diagnosis		Total
	ILD	Non-ILD	
ILD	32	13	45
Non-ILD	3	32	35
Total	35	45	80

## Discussion

In our study, the mean age of patients was 58 years, however interstitial lung disease was seen in adults as well as children. No significant gender wise differences were seen with the male:female ratio of test subjects being 0.95:1. However, a variability in gender profile based on the underlying disease has been reported in literature. Lim et al. reported an equal male-to-female ratio in idiopathic pulmonary fibrosis, collagen vascular disease associated pulmonary fibrosis and hypersensitivity fibrosis but a female dominance in sarcoidosis and male dominance in pneumoconiosis in their study [9]. In another study from a tertiary care centre in India, Gagiya et al. reported a male dominance (66.5%) but Kumar et al. in another study from India reported female dominance (54.64%) [10,11].

Smoking is an identifiable risk factor in more than two-fifth cases of RBILD, desquamative interstitial pneumonia (DIP) and pulmonary Langerhans-cell histiocytosis (PLCH). The dominance of smokers in present study also endorsed an increased risk of ILD in smokers as also observed by Jin et al. [12].

Breathlessness was the most common complaint (37.5%) and dyspnoea (27.50%), shortness of breath (25.0%) and progressive dyspnea (10%) were the chief complaints. Compared to our study, Gagiya et al. in their study, had reported breathlessness on exertion (100%), dry cough (43.29%), anorexia (50%) and joint pain (16.65%) as the presenting complaints [10].

**Figure 1:**
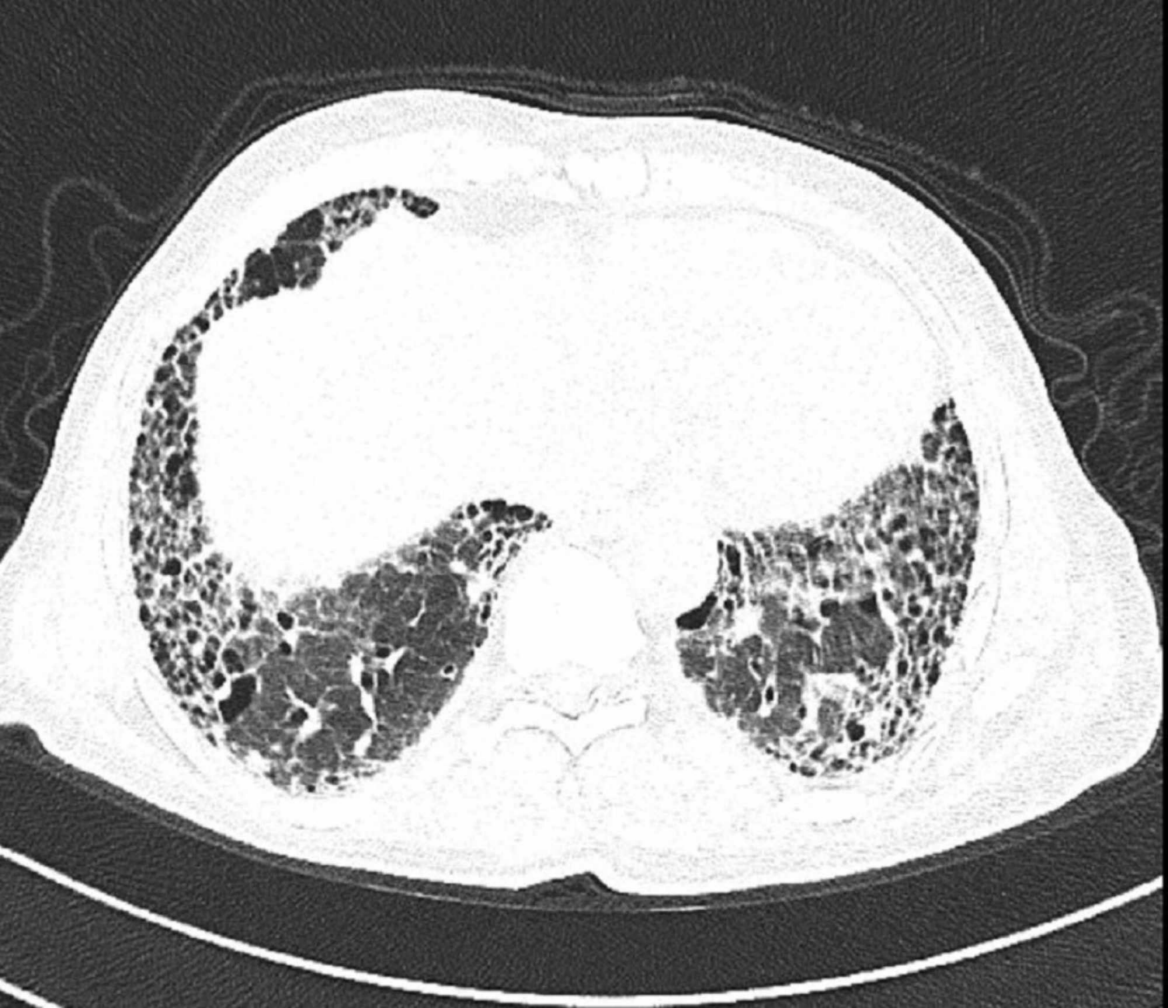
Honeycombing pattern in UIP UIP: Usual interstitial pneumonia

ILD was recognized by apicobasal gradient of subpleural honeycombing, bronchiectasis, reticulation pattern. Honeycomb pattern on HRCT is highly suggestive of usual interstitial pneumonia (Figure 1).

**Figure 2:**
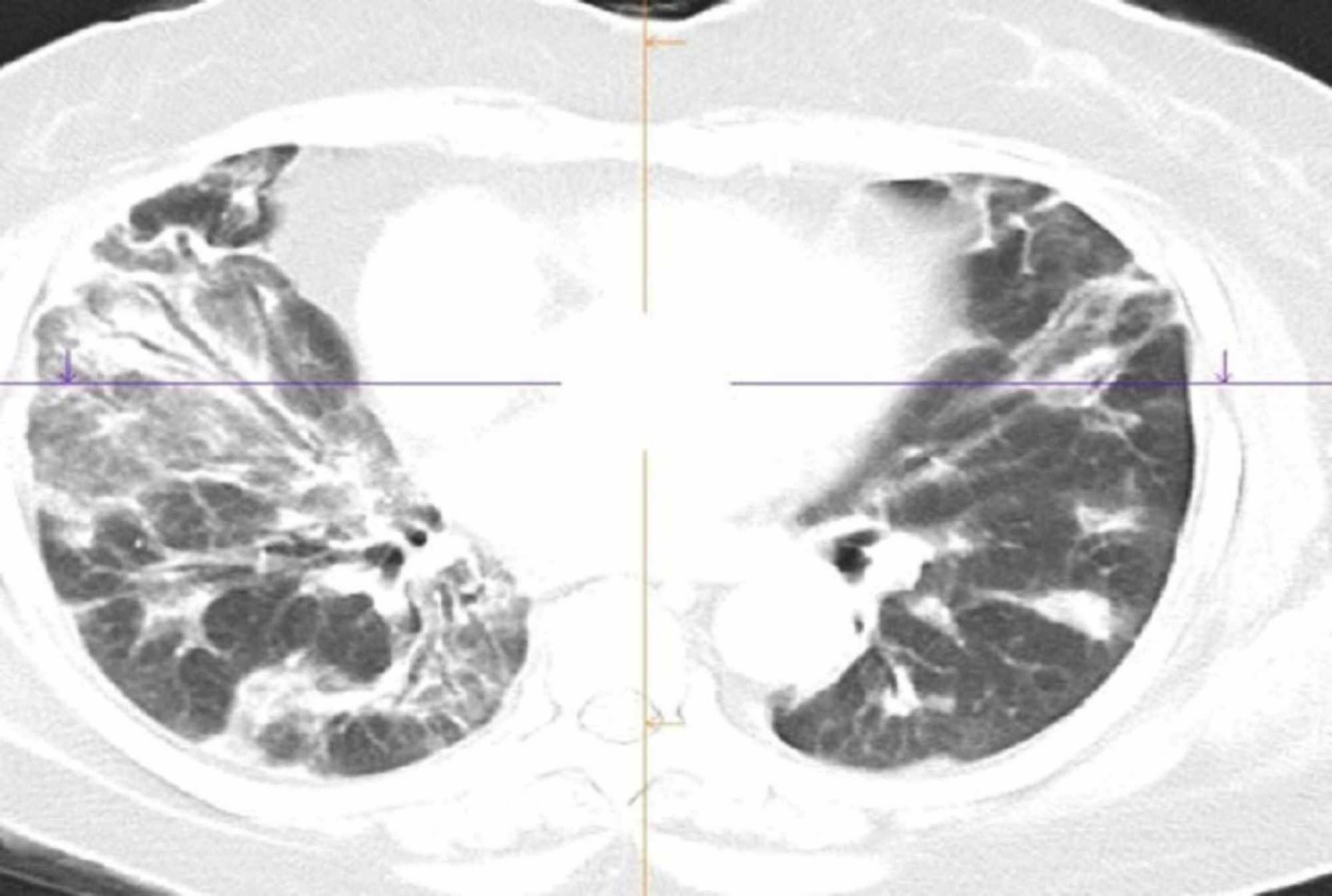
MDCT axial view of thorax showing bilateral ground glass attenuation with areas of consolidation MDCT: Multidetector computed tomography

Cryptogenic organizing pneumonia (COP) was found to be the next most common interstitial lung disease. The principal findings were ground glass opacities and reticulation involving predominantly subpleural and basal lungs. Nodular pattern was random, and thus provided a highly suggestive diagnosis of COP (Figure 2). Traction bronchiectasis was noted in all these patients.

We did not find honeycombing pattern in any of the COP cases as observed by Lee et al. in some of the cases in their series [13]. There were four cases with the “atoll” or reversed halo sign (a central ground-glass opacity surrounded by a crescent or ring of consolidation) which is found in COP, but can be seen in other conditions like chronic eosinophilic pneumonia (CEP), paracoccidioidomycosis or tuberculosis [14,15].

**Figure 3:**
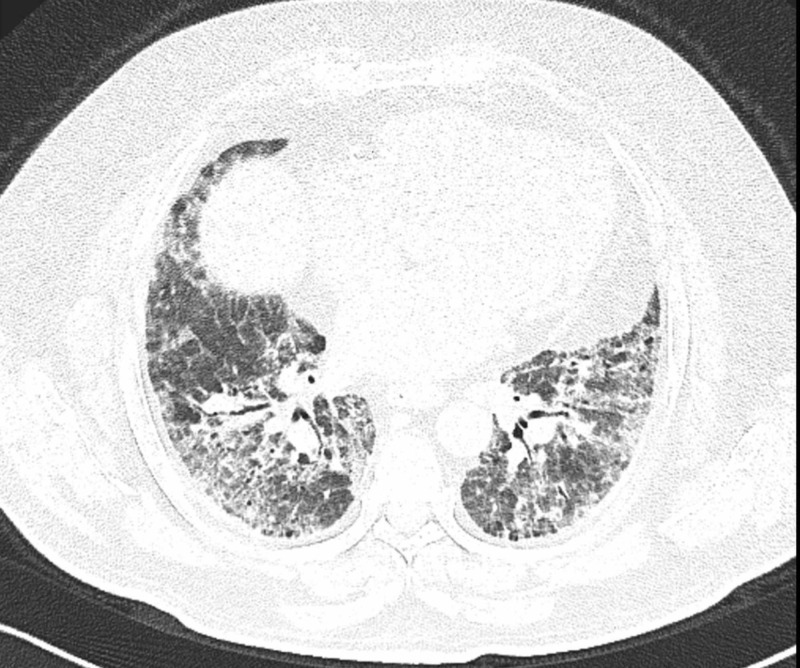
MDCT axial view showing bilateral basal ground glass opacity and reticulation pattern MDCT: Multidetector computed tomography

Third most common ILD diagnosis was non-specific interstitial pneumonia (NSIP). It was recognized by ground glass opacity, irregular areas of consolidation, irregular linear opacity, bronchiectasis, absence of reticulation, absence of honeycomb pattern and centrilobular nodularity (Figure 3) [16,17].

On final diagnosis, ILD was confirmed in 35 cases. Out of the 35 cases confirmed as ILD in final diagnosis, 32 were also confirmed as ILD by MDCT whereas among remaining 45 non-ILD cases as per final diagnosis, 32 were confirmed as non-ILD by MDCT. Thus, for detection of ILD, MDCT had 32 true positive, 13 false positive, three false negative and 32 true negative cases and correspondingly, it was found to have a sensitivity of 91.4% and specificity of 71.1%. The positive predictive and negative predictive value of MDCT for detection of ILD was 71.1% and 91.4%, respectively. Overall diagnostic accuracy was 80%.

The findings of present study thus showed that MDCT is a useful modality for detection and differentiation of ILD into different subtypes. It was also revealed that apart from MDCT imaging features, patient’s clinical history also helps to improvise the accuracy of results.

## Conclusions

The findings of present study concluded that MDCT is a useful modality for detection of ILD and its spectrum in suspicious cases, however, clinicopathological correlation and history taking is essential in order to obtain a more accurate and precise diagnosis. MDCT as a single modality had a high sensitivity for detection of ILD and could be recommended as first line diagnostic imaging technique.
